# Model evidence for distinct origins of glacial–interglacial and millennial signals in Greenland dust

**DOI:** 10.1073/pnas.2531908123

**Published:** 2026-03-30

**Authors:** Peter O. Hopcroft, Denis-Didier Rousseau

**Affiliations:** ^a^School of Geography, Earth, & Environmental Sciences, University of Birmingham, Birmingham B15 2TT, United Kingdom; ^b^Institute of Physics-Centre for Science and Education, Division of Geochronology and Environmental Isotopes, Silesian University of Technology, Gliwice 44-100, Poland; ^c^Geosciences Montpellier, Université de Montpellier, Montpellier Cedex 05 34095, France; ^d^Lamont Doherty Earth Observatory, Columbia University, Palisades, NY 10964

**Keywords:** Last Glacial Maximum, earth system model, abrupt climate change, mineral dust, paleoclimate

## Abstract

Greenland ice-core records show that the mineral dust flux during the last glacial maximum was twenty times greater than present and it responded rapidly during North Atlantic abrupt climate events. Both glacial–interglacial and rapid modes of dust variability have been suggested to originate from changes in storminess over Asian deserts. However, geochemical measurements do not rule out sources in Africa and Europe, and rapid changes in the source strength over Asia are yet to be unambiguously identified in North Pacific dust flux records. With a suite of Earth System model simulations of the last glacial period we show that latitudinal rainbelt shifts characteristic of abrupt events may have had an important impact on dust across the Atlantic region. Unlike for the glacial–interglacial difference, these faster, decadal to centennial scale transitions drive significant dust generation and meridional dust transport which enhances dust delivery over Greenland. These results help reconcile several features of the glacial dust cycle and thereby question the paradigm that Asian desert conditions can explain both modes of variability in Greenland dust records. The model points to a greater sensitivity of the poleward dust transport to climate change and provides a basis for investigating the potential role of dust radiative feedbacks during abrupt climate change.

## Significance

Ice-cores provide among the clearest high-resolution records of Earth’s climate for the past thousands of years. Measurements of natural dust flux from Greenland ice-cores show very large amplitude and rapid variations that take place on a timescale of decades to centuries. These have largely been interpreted as arising from changing storminess over deserts in Asia. In simulations of the last ice-age with three-dimensional numerical models we show that this picture is incomplete and that dust was also sourced from Africa, especially during the coldest (and dustiest) phases. This modeled response reconciles several seemingly contradictory observations of the ice-age dust cycle and thereby challenges the paradigm that Greenland records respond primarily to conditions over Asian desert regions.

During the last glacial maximum (LGM) from around 26 to 19 thousand years B.P. (kyr BP) (1) Earth’s climate was around 4-6 °C cooler than today (e.g., refs. 2, 3, 4). Huge ice-sheets covered parts of North America and Europe and greenhouse gas levels were much lower than during the preindustrial (5). Terrestrial, ice, and marine sediment cores also show that this cooler climate was significantly dustier than present (6–8) which is consistent with the climate being less favorable to vegetation (9).

The last glacial period was also very different from the Holocene because of the repeated occurrence of abrupt climate change events (e.g., refs. 10 and 11). Throughout the last glacial period, reconstructions show Dansgaard–Oeschger (D-O) cycles characterized by abrupt warming events over Greenland (12) each followed by a more gradual return to colder (stadial) conditions (13). D-O events are thought to be an intrinsic oscillation of the system that manifests during glacial periods (e.g., refs. 14 and 15). In some D-O stadials there is evidence for massive discharges of icebergs into the North Atlantic (16). These Heinrich stadials (HS) are less clearly expressed at high-latitudes (17), but register as major hydrologic events over the tropics (e.g., 18).

Ice-core records show that the LGM dust flux increase was particularly large over both poles, reaching around 20 times preindustrial levels over Greenland (19, 20) [where this ratio already accounts for synchronous rapid changes in snow accumulation at the ice-core sites (e.g., refs. 20, 21, 22)]. During D-O warmings the dust depositional flux reduced rapidly over Greenland approximately synchronously with other ice-core parameters (23, 24). During HSs the dust depositional flux over Greenland increased by as much as a factor of 6 to 10 (25, 26). Dust records from around the globe also show abrupt changes particularly over Europe (27, 28) and the tropical Atlantic (e.g., refs. 29 and 30). However, Greenland ice-core records provide key benchmarks for unraveling the mechanisms of abrupt climate change here broadly defined as decadal to centennial timescale shifts in regional conditions (24, 31, 32) because of the high temporal resolution across multiple parameters and several events (33). Dust is an integral component of the abrupt climate record and so it is therefore critical to better understand the causes of the large amplitude Greenland dust fluctuations.

The LGM dust flux increase over Greenland has been attributed to an intensified production of dust over Asian deserts by both geochemical analyses of ice-core dust (34–36) and reduced order modeling (33). The paradigm relies on a major increase in storminess over Asia during colder periods, perhaps driven by large-scale changes in the latitudinal temperature gradient. Although the amplitude of changes is important, a potential difference in the underlying mechanism of the rapid versus long-term glacial–interglacial dust flux change over Greenland has not been studied in detail. Hence, the rapid changes in dust across stadial–interstadial transitions are also widely held to be controlled by storm activity over Asian deserts (25, 33, 37) and this is thought to explain the very rapid response of dust over Greenland (23) whereby most recorded transitions take place over around 30 to 100 y (24).

However, other records do not unambiguously support this paradigm. The North Pacific marine sediment core dust flux record from site SO202-7-6 shows changes in local dust flux of the order of 10 to 20% during abrupt events (22) which is potentially supported by other records (38–40) although many do not have adequate temporal resolution. In contrast the dust flux change over Greenland approaches a factor of 8 difference (26). Moreover, a one-to-one relationship between Asia and Greenland (e.g., ref. 25) is also not evident when examining a wider selection of Asian loess records (41–43). In contrast, Greenland dust variations were highly temporally correlated with dust activity over Europe (27, 28) and newer geochemical analyses of dust samples from Greenland stadial phases do not rule out Europe and/or North Africa (44–47) as important source regions.

Three-dimensional Earth System models show that LGM Greenland dust flux is sensitive to changes in the land-surface due to lower atmospheric CO_2_ and a dryer climate (9, 48, 49). These factors cause simulated deserts to expand, leading to globally increased dust emissions. However, models are also largely dependent on the inclusion of glaciogenic (i.e., nondesert) dust source regions in order to produce large enough changes in dust (48, 50, 51). This means that the simulated increase in polar dust fluxes in several models are dominated by expansion of emissions zones rather than increased gustiness (e.g., refs. 48 and 50).

Although it remains unclear to what extent models can reproduce abrupt Greenland dust changes (51, 53), coupled model simulations can reproduce some key features of the climatic changes across of these events (54, 55). These simulations agree that the hydrological cycle around the Atlantic is particularly sensitive to abrupt climate changes (e.g., refs. 56, 57, 58) which is in broad agreement with records (e.g., refs. 59, 60, 61, 62). Given a strong coupling between hydrology and both the generation and below-cloud scavenging of dust, we therefore propose that glacial–interglacial and millennial scale changes in Greenland dust result from different regional processes. We analyze a suite of atmosphere-dust simulations to evaluate this hypothesis and to better understand potential radiative feedbacks for different timescales of climate change.

## 1. Results

**Fig. 1.**
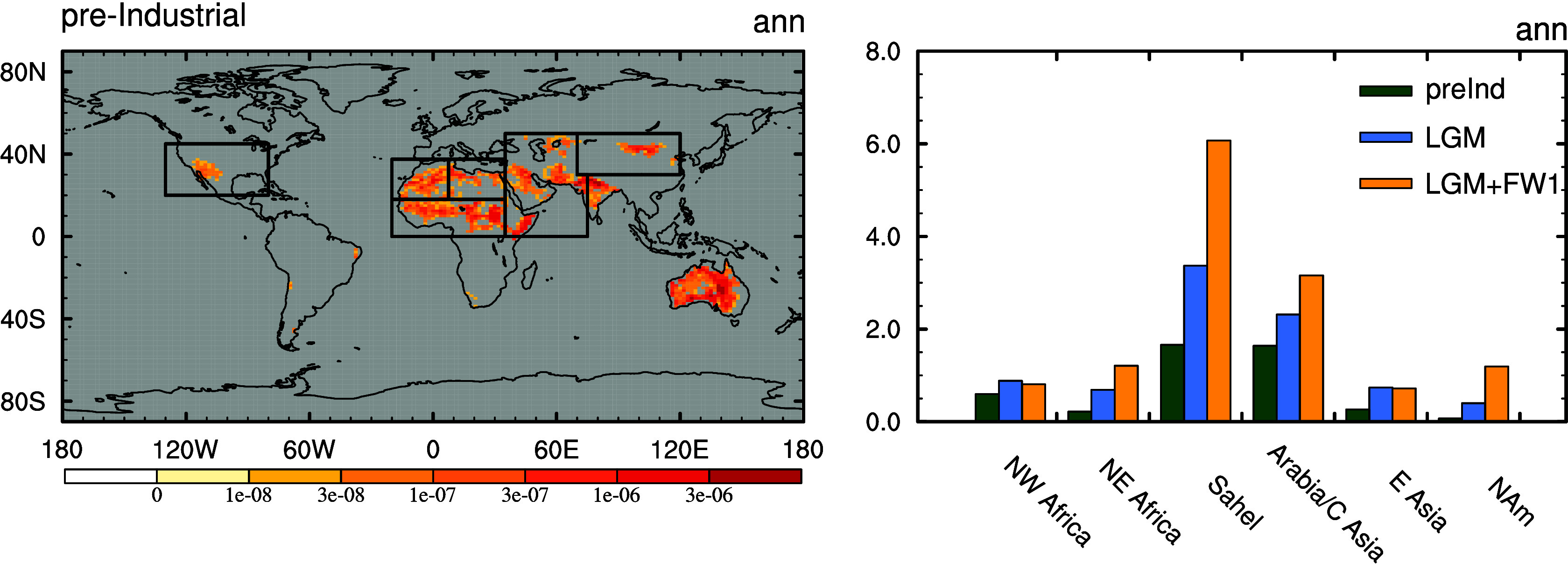
*Left*: HadGEM2 simulated annual-mean dust emission in the preindustrial simulation summed over particles in size bins 1 to 3 (≤1μm radius). with six Northern Hemisphere regions following DustCOMM (e.g., ref. 52) and as indicated by black boxes; *Right* the summed changes over these regions for the preindustrial, LGM and LGMfw1 simulations.

**Fig. 2.**
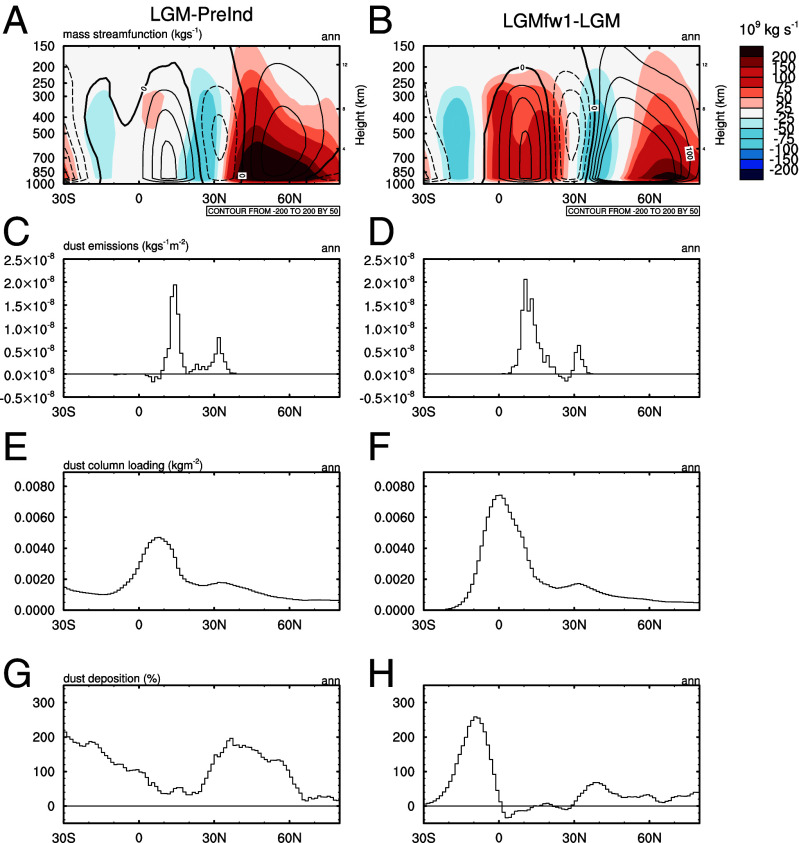
Potential relationship between atmospheric and dust changes over the North Atlantic sector. Annual-mean (*A*) preindustrial and (*B*) LGM atmospheric mass streamfunction (contours in kgs^−1^) and streamfunction anomalies (shading, anomalies as per titles); (*C* and *D*) dust emission anomalies; (*E* and *F*) column-integrated dust loading anomalies; and (*G* and *H*) relative (%) anomalies in dust deposition anomalies. All variables are averaged over 60 °W to 20°E and dust fields are integrated over bins 1 to 3 corresponding to particles sized 0.03 to 1.0 μm radius.

Earth System model simulations of the preindustrial, LGM and LGM with a North Atlantic cooling, LGMfw1 (see *Materials and Methods* for setup) are compared in the following. Fig. 1 shows the simulated response of annual-mean emissions across different regions and Fig. 2 compares the response of the atmospheric streamfunction, dust emissions, column loading, and deposition across the Atlantic sector. Both figures are restricted to the finer particles (<1 μm radius) because these are capable of reaching far-field sites like Greenland (see, e.g., figure 2 of ref. 63). There is a widespread increase in emissions in the LGM (Fig. 1 and *SI Appendix*, Fig. S1). This is driven by expansion of bare soil areas, reductions in rainfall in some regions and changes in local wind speeds. For example, over Asian deserts the bare soil fraction expands for the LGM, the mean wind speeds increase and precipitation is reduced (as shown in *SI Appendix*, Fig. S2). Over the Pacific the depositional flux increases 2.6-fold in the North West Pacific (*SI Appendix*, Fig. S1) which is comparable but smaller than the reconstructed fivefold increase recorded at site SO202-7-6 (22).

Elsewhere, over Africa, there are some major increases in emissions but the simulated LGM circulation does not favor export of this dust northward (see also further analysis of atmospheric transport below). As a result the model produces a relatively modest doubling of the flux over Greenland. This is much smaller than the reconstructed 20-fold increase typical of Greenland ice-core records (25). This underestimation is common to other models (e.g., refs. 9, 64, and 65).

Figs. 1 and 2 also show how an abrupt cooling imposed over the North Atlantic (in response to an idealized 1.0 Sv freshwater forcing; see *Materials and Methods*) reorganizes both the atmospheric circulation and dust cycle relative to the LGM simulation. This simulation shows a substantial increase in the strength of the northern hemisphere Hadley Cell over the tropical Atlantic (as shown by the significant positive anomalies in the mass streamfunction) which is consistent with idealized modeling of hemispherically asymmetric thermal anomalies (*SI Appendix*, Fig. S2) and conceptually, is a direct result of the resultant hemispheric energy imbalance (66). This result is also very similar to other models (e.g., ref. 67). This enhanced circulation intensifies northward dust transport. Accompanying this there is a huge increase in dust emissions (spatially decomposed in Fig. 1) and hence loading over the tropical Atlantic and Africa (Fig. 2 and *SI Appendix*, Fig. S1).

Over the region 0 to 35°N by 80°W to 40°E, dust emissions, tropospheric loading (summed to 10 km altitude), and total dust deposition change by +65%, +54%, and −14% respectively for the <1 μm particles (*SI Appendix*, Table S1). Deposition as a proportion of column loading in this region is reduced by 45%. This large change is mostly due to the southward shift of the more intense precipitation band (shown in *SI Appendix*, Fig. S2) which leads to a substantial reduction in wet-deposition of dust. Together these changes enable the land-masses around the northern tropical Atlantic to act as an extremely efficient dust exporter. These changes are proportionally similar when the full particle size range is used, except that the deposition increases. This is because of the disproportionate influence on the dust mass budget of the largest particles which mostly resettle close to the sources’ regions in this model (68–70). Equivalent simulations that include glaciogenic source regions are compared in the Supporting Information *SI Appendix*, Fig. S3 and these show qualitatively similar responses as described above, with the mid-latitudes (including Europe) being much dustier (*SI Appendix*, Figs. S1 and S3).

The large changes in dust also have a major regional impact on the radiation balance. For the LGM relative to the preindustrial there is a widespread negative forcing of −1.8 W m^−2^ (averaged over the region 0 to 35°N by 80°W to 40°E) leading to additional regional cooling (49, 51). In response to the freshwater forcing, the radiative forcing change is substantially larger and averages −3.6 W m^−2^ over the same region, see *SI Appendix*, Fig. S4.

**Fig. 3.**
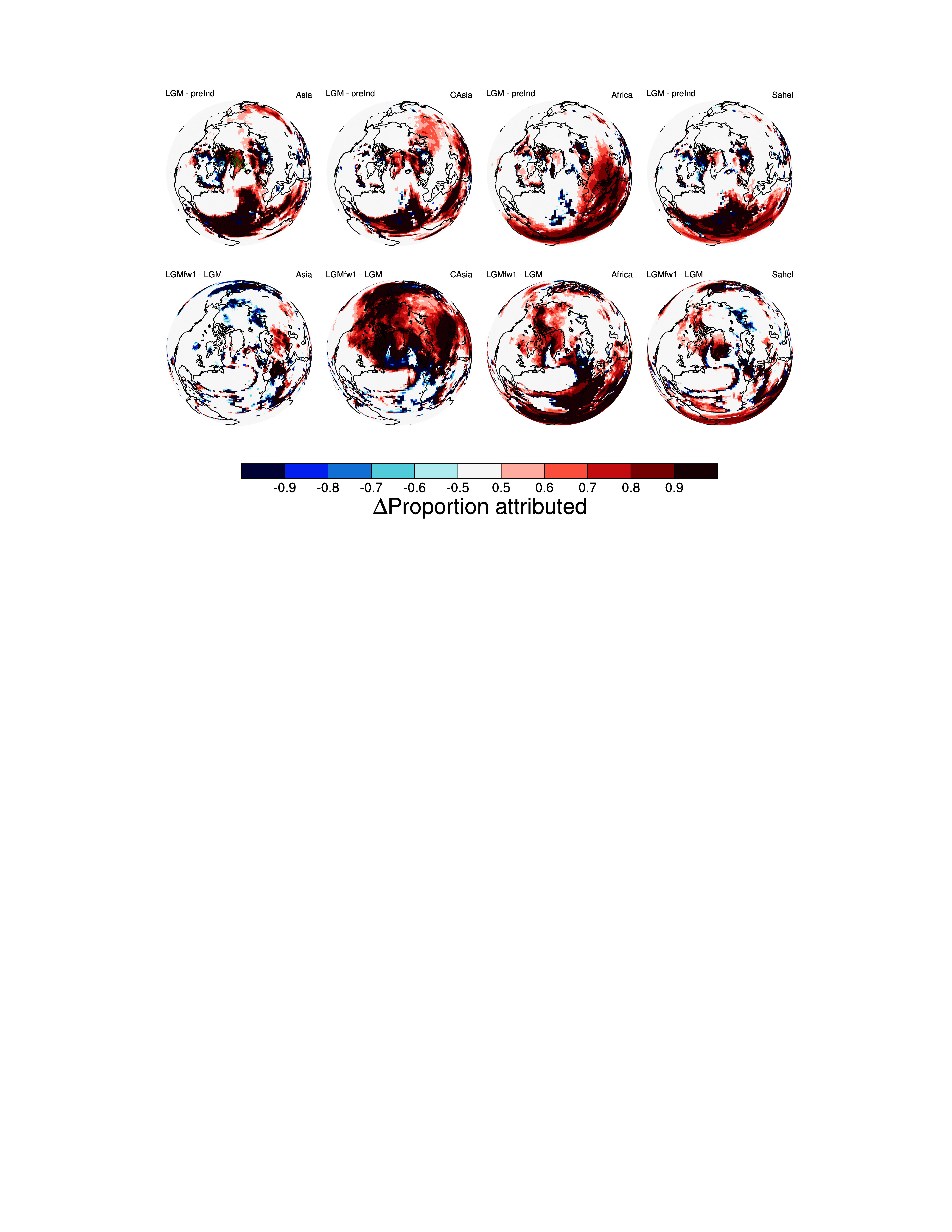
Simulated fractional contribution to the dust depositional flux change over the Northern Hemisphere and attributed to sources in East Asia, Central Asia/Arabia, Northern Africa, and the Sahel for the LGM - pre-Industrial (top row) LGMfw1 - LGM (lower row). The results are for the ≤1.0 μm radius in accordance with the predominance of finer particle content of ice-core dust. Ice-core sites [NEEM (33), NGRIP (25), GISP2 (19) and Renland (71)] are shown by green symbols in the first panel.

Separating the emissions by region (*Materials and Methods*) allows us to fingerprint the changes simulated over Greenland (Fig. 3). East Asian dust source regions emerge as the major contributor of the glacial–interglacial signal in the model (Fig. 3). This is in agreement with a wealth of observational data (e.g., refs. 35 and 36). However, this region is not a key driver of the abrupt dust signal (LGMfw1 - LGM). In this case Africa and Arabia and Central Asia are much more significant which is consistent with the simulated changes in emissions (Fig. 1) and deposition discussed above. However, we note that central Asian dust is not supported as a source of Greenland dust (46). We also find a very similar response in simulations including glaciogenic sources in North America, Siberia, Alaska, and Europe, or with a much weaker cooling prescribed in the North Atlantic (forced with a freshwater input of only 0.4 Sv), see *SI Appendix*, Fig. S5, suggesting the underlying mechanisms are robust over a range of conditions. Whether the enhanced dust derived from Africa is sourced from the Sahara or further south in the Sahel is addressed in further sensitivity simulations. Although the emission increase more over the Sahel (Fig. 1) it is the enhanced circulation (as shown in Fig. 2) that is responsible for delivering dust from the Sahara that contributes to the signal further North and over Greenland. This is also consistent with observational geochemical evidence that appears to rule out the Sahel as a major source of Greenland stadial dust (e.g., ref. 46). However, there is considerable spatial variability in the isotopic and elemental signature of North African dust; see *SI Appendix* for further discussion.

**Fig. 4.**
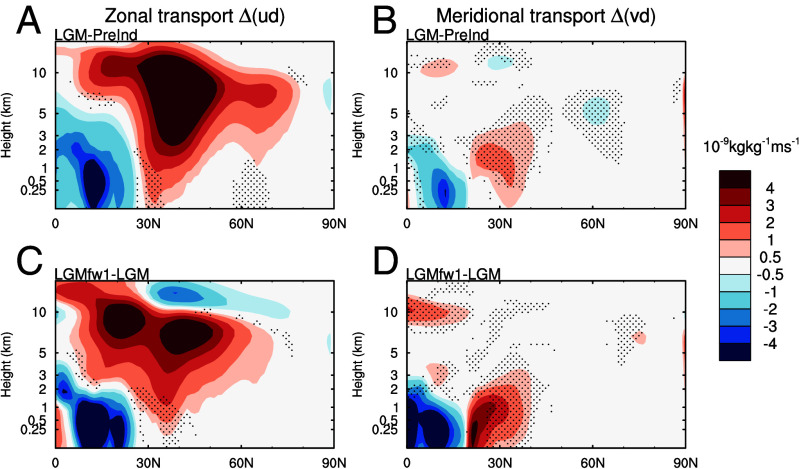
Dust transport anomalies (10^−9^ kg kg^−1^m s^−1^: i.e., dust mixing ratio [kg kg^−1^] multiplied by wind velocity [m s^−1^]) for LGM-pre-Industrial (*A* and *B*), and LGMfw1-LGM (*C* and *D*). Colored shading shows the total transport term, stippling indicates where eddies contribute at least half of the total change. Anomalies are calculated over the region from 80°W to 20°E). *A* and *C* show zonal (East-West) anomalies and *B* and *D* how meridional (South-North) anomalies.

Given the potential significance of the circulation changes, we further decompose the simulated dust transport into changes in the mean and transients using archived daily model fields. Considering the *LGM* minus *pre-Industrial* anomaly, transport changes are dominated by the zonal (East-West) response (Fig. 4). This is mostly due to increased emissions from Asia (and North America) and the subsequent entrainment into the mean circulation, rather than a strong augmentation in the mean circulation. Across the Atlantic sector, the anomalous meridional transport (vd) shows a combination of enhanced eddy-driven circulation between 20 and 40°N and reduced northward transport in the mid-latitudes between 40 and 70°N. In combination this does not lead to a clear enhancement of meridional dust transport.

For the abrupt north Atlantic cooling simulation (LGMfw1 relative to LGM) there is a similar change in the zonal transport from Asia and North America, but with a much greater increase in the northward transport due to transient eddies over the North Atlantic between 20 and 40°N that extends upward from the boundary layer and is located directly under the strengthened descending branch of the northern Hadley Cell shown in Fig. 2*B*. This latter feature is driven by the enhanced atmospheric overturning in response to the cooling imposed over the North Atlantic. This activates a new transport pathway from the latitudes of the Sahara to the mid-troposphere over the extratropics and poles (Fig. 4).

Across most of the Atlantic sector the changes induced by the stadial cooling (LGMfw1-LGM) are larger than the equivalent LGM - pre-Industrial anomalies. This thereby implies a much greater sensitivity of the meridional dust transport on shorter abrupt timescales than previously assumed in this context. This difference is attributable to the more localized forcing on the dust cycle caused by the North Atlantic cooling and the subsequent interhemispheric energetic imbalances (e.g., refs. 72 and 73).

## 2. Discussion

We have shown that glacial–interglacial (LGM - PreInd) and millennial-scale (LGMfw1.0 - LGM) simulations show very different fingerprints of dust cycle response. The glacial–interglacial signal is dominated by changes in sources, particularly over Asia which today represents a major source of dust to Greenland (74). The north Atlantic cooling signal is characterized in the model by a major disturbance of the dust cycle over the land masses surrounding the Atlantic basin, with huge changes in dust emissions, lifetime, and transport all connected to the southward shift of the tropical rainbelts (51).

There are three key features seen in recent studies that can be explained by enhanced meridional dust transport from the land masses surrounding the tropical Atlantic. Millennial scale changes in dust properties have been recorded in the Sea of Japan sediments (75) but a more direct record of dust flux over the Pacific shows very little change across abrupt climate events (22). This muted response over the Pacific Ocean is consistent with the simulations where the simulated dust flux changes in response to North Atlantic cooling are of the order of 20 to 50%. A tropical, meridional model of dust delivery to Greenland could additionally account for the synchronous nature of abrupt dust variations in Europe and Greenland (27, 28) and recent geochemical evidence of a remote dust source over Europe that was active during cold-phases (Greenland stadials) (76), although only if dust deposition in the two regions is linked by a common long-transport route. The simulated tropical dust source can also satisfy newer geochemical source attributions for peak Greenland fluxes. Inferences from lead and strontium (45) and hydrogen, strontium, neodymium, and hafnium isotopes (46) and other elements (47), all suggest Europe and/or North Africa as potential sources of Greenland ice-core dust during peak (cold-climate or stadial) dust phases.

The model simulations have some drawbacks in representing North Atlantic abrupt events. The forcing employed here is most likely too large from both a glaciological standpoint (77) and from the comparison of simulated dust changes in the tropics (51). The forcing with meltwater also means that the simulations are unlikely to be fully representative of D-O oscillations (78). These aspects could be addressed by incorporating dust feedbacks into simulations that are able to produce spontaneous D-O -like oscillations (e.g., refs. 54 and 79) or where realistic meltwater fluxes are prescribed (80).

Nevertheless, the underlying mechanisms discussed here are largely independent of these more detailed considerations. This is because evidence from a range of climate models shows that tropical rainbelts are highly sensitive to changes in North Atlantic temperature gradients (81) and thus both the meridional and gyre circulations. A southward shift in the intertropical convergence zone (ITCZ) has been inferred from many records (18, 59) and in multiple different climate models (56, 81) and has been previously invoked in the case of dust (28). Additionally, repeating these simulations with a weaker cooling over the North Atlantic, results in qualitatively very similar conclusions.

However, the modeled LGM dust cycle is also imperfect despite the inclusion of glaciogenic source regions. One key metric is the LGM divided by preindustrial depositional amplification factor. This can be calculated from dust archives and compared with model simulations. A compilation of preindustrial and LGM dust records (82) shows a very large increase in dust over both poles (*SI Appendix*, Fig. S6) but this is not replicated in this or other models. It remains unclear whether this is related to source or sink processes (e.g., ref. 83) or how an improved simulation of this aspect would alter the sensitivity to abrupt climate signals.

The radiative effects of dust are also highly dependent on the prescribed optical parameters (e.g., refs. 84 and 85). HadGEM2-ES, like many models, relies on observations from the Sahara but regional variations in the mineralogy can be important. Moreover, the dust events are necessarily more-or-less entirely parameterized in these global models which means they are sensitive to representation of turbulence among other processes and how these respond to climatic change. Very high-resolution modeling would be needed to address this aspect.

## 3. Conclusions

Greenland ice-core records show that the dust flux varied significantly during the last glacial period. The underlying causes of the different timescales of variation remain poorly understood. Although many early geochemical analyses of the ice-core dust records imply a highly variable but dominant Asian source during the glacial period, other paleo-records do not unanimously support this, either showing correlation with Greenland (25), a significant lag (41–43), or only a muted signal (22). More direct dust flux records from Europe (28) and Atlantic (30) generally show many of the same features observed in Greenland yet with a lower amplitude. We show with ESM simulations that the majority of the additional dust reaching Greenland for the LGM is indeed sourced over Asia but that in response to abrupt Atlantic cooling, significant additional dust comes from regions around the Atlantic, with relatively less of the signal originating from Asia. We therefore propose that the millennial and glacial–interglacial signals observed over Greenland have distinct origins, something that has not been considered directly in previous work (27, 28) but is consistent with findings discussed above. The model results also imply a much greater meridional transport of dust than implied by the existing paradigm and therefore higher overall sensitivity to abrupt climate changes. This distinct origins hypothesis needs further testing with independent models and paleo-records, particularly in higher-resolution models capable of spontaneous D-O-like oscillations but could provide a basis for better capturing dust radiative feedbacks during abrupt climate events.

## 4. Materials and Methods

### 4.1. Paleo-Dust Records.

All ice-core dust records (19, 25, 33, 71) are referred to in terms of fluxes that have been corrected for past variations in snow accumulation in order to separate the changes in dust deposition from the precipitation variations (20, 21).

### 4.2. Earth System Model.

Simulations are performed with the Earth System model HadGEM2-ES (86, 87) which was developed at the Met Office and used extensively in the Coupled Model Intercomparison Project 5 (CMIP5) (88). This model has a horizontal resolution of 1.875^°^×1.25° (in longitude-latitude) with 38 unequally spaced vertical levels reaching to around 37 km altitude. HadGEM2-ES is among the better performing models in CMIP5 (e.g., ref. 89, their figure 2). Here, we use the atmosphere-only version of the model termed HadGEM2-A which includes dynamic vegetation and aerosols but not interactive atmospheric chemistry.

A key benefit of HadGEM2 is the inclusion of an interactive mineral dust cycle that is coupled to the atmosphere, including the radiation scheme, the land-surface, and to the model’s dynamic vegetation (90, 91). Dust is represented with six atmospheric traces with size bin boundaries at 0.0316, 0.1, 0.316, 1.0, 3.16, 10.0, and 33.16 μm particle radius. Dust emissions are calculated every time-step as a function of bare soil fraction, soil moisture, and wind speed following ref. 92. Six dust atmospheric tracers are then subject to gravitational settling and below-cloud scavenging. Particles are assumed to be spherical and their radiative impacts are represented with Mie theory (90) and optical properties from ref. 93. HadGEM2 is able to reproduce the major features of the observed dust cycle (49, 91, 94) and this dust model is the basis of that used in the more recent UK Earth System model (68).

### 4.3. Paleoclimate Simulation Setup.

For the LGM, HadGEM2-A was forced with reconstructed ice-sheet area, topography, and sea-level (95), greenhouse gas concentrations from ice-core records (96), and Earth’s orbital parameters (97). The model is run in atmosphere-only configuration with monthly sea-surface temperatures (SSTs) and sea-ice distribution prescribed from simulations of the LGM with a slightly lower-resolution coupled atmosphere-ocean model HadCM3 (98). In the default CMIP5 configuration, the HadGEM2 LGM simulation produced a dust-bowl like climate (99). In this and previous studies we use updated parameters in the land surface that allow for a stable climate state for both the preindustrial and the LGM (49, 99).

In the LGM simulations, we follow the approach of refs. 50 and 100 and introduce glaciogenic dust sources in Europe, North and South America, and Siberia to represent additional erodible material resulting from widespread erosion by glaciers during the LGM. This glaciogenic simulation is labeled LGMglac as described previously (51). The glaciogenic LGM simulations were also run under stadial-like conditions by employing different SSTs and sea-ice which were calculated from coupled LGM simulations with HadCM3 forced with a freshwater input of 1 Sv (=10^6^ ${\text{m}}^{3}\;s^{-1}$) for 100 y over the North Atlantic. This simulation is labeled LGMglac+fw1 (51). We also test the impact of weaker North Atlantic cooling for which only 0.4 Sv is added to the North Atlantic (LGMglac+fw0.4). The simulations are run for at least 50 y with the final 30 used for calculating climatologies. The main simulations are listed in *SI Appendix*, Table S2.

### 4.4. Restricted Source Simulations.

To constrain the sources of dust arriving at Greenland we reran all of the simulations described above but with dust sources prevented from one of several regions thought to be important for the dust cycle today and/or in the past. These are Asia, Arabia, and Central Asia, North Africa, the Sahel (0 to 20°N), and Europe. These regions mostly follow those defined in DustCOMM (e.g., ref. 52) and are indicated by black boxes on Fig. 1. The resultant difference between the full global-sources run and the restricted-sources simulation is attributed to that region.

In many cases the simulated emissions differ from the equivalent emissions in the global run. This is because of interannual variability and because of the missing radiative effects from dust that are omitted when emissions from key regions are deactivated and the subsequent changes in regional climate conditions. The emissions differences are given in *SI Appendix*, Table S3. This demonstrates that the model has a nonlinearity in terms of how the total dust response is related to emissions in separate regions. To account for this we find the 25 y from the restricted-sources run for which the total emissions over the Northern Hemisphere (NH) are closest to emissions integrated over the same area in the full model simulation (but omitting the region of zero emissions). For example, in the simulation with emissions deactivated over Africa, the emissions are summed over the Northern Hemisphere except for North Africa and the 25 y with best agreement with the equivalent spatial sum in the global-sources run are used to calculate depositional fluxes.

### 4.5. Radiative Forcing Calculation.

In order to calculate the dust radiative forcing, the model is run in what is referred to as double-call mode. In this the radiation scheme is called an additional time at every timestep and in this call the radiative effects of dust are not included. The difference with the default radiation calculation is then archived. These diagnostics are here used to calculate the net (short-wave plus long-wave) forcing at the top of the model. This resultant forcing is highly dependent on the relative proportions of fine vs. larger particles (70) and the scattering and absorption parameters used (84, 85) but they give a broad indication of the potential impacts on the climate system. Although in general increased dust loading equates to a net negative radiative forcing there are regions that show the opposite. These regions of positive radiative forcing are caused by a coincident change in the underlying land surface. In the case of the LGM relative to the preindustrial, areas of new land (e.g., the Red Sea) or ice-cover (e.g., North America), can lead to a weak net positive forcing. With more detailed calculations the land surface and aerosol effects can be effectively separated and this is discussed in more detail elsewhere (49). At the global scale, this correction is relatively minor.

### 4.6. Dust Transport Decomposition.

HadGEM2 does not include source tagging of dust tracers. The transport of dust by the atmospheric circulation must instead be calculated from the archived wind speeds and dust mixing ratios. For this purpose we archived daily-mean model outputs of dust and wind speeds at all model levels and calculated the relative contributions of the mean circulation and stationary and transient eddies. Higher frequency outputs could be used (e.g., 6-h) but we opted to use daily means as a compromise between fidelity of the transport calculations and compute storage required.

The mean transport of dust ($\bar{\mathrm{vd}}$), where $\bar{x}$ indicates time-mean, d is the dust mass mixing ratio (kg kg^−1^) and v is the meridional wind (ms ^−1^) can be decomposed [following Peixoto and Oort (101)] as

[1] $$\begin{array}{cc} {}[\bar{\mathrm{vd}}]= & \left[\bar{v}\right]\left[\bar{d}\right]+\left[\bar{v^{*}}\bar{d^{*}}\right]+\left[\bar{v^{\prime }d^{\prime }}\right],\\ = & \text{mean}\text{+ stationary eddy}\text{+ transient eddy}, \end{array}$$

where v’ are the departures from the monthly mean, [v] denotes the zonal mean, and v^∗^ is the departure from the zonal mean.

The change in the dust transport between two simulations can then be decomposed into the following terms:

[2] $$\begin{array}{cc} \Delta [\bar{\mathrm{vd}}]= & \Delta \left(\left[\bar{v}\right]\left[\bar{d}\right]\right)+\Delta \left([\bar{v^{*}}\bar{d^{*}}]\right)+\Delta \left([\bar{v^{\prime }d^{\prime }}]\right). \end{array}$$

The zonal transport can be decomposed in an analogous manner by replacing v with the zonal wind speed, u. The shading on Fig. 4 is calculated for grid cells where the sum of the stationary and transient eddy terms greater than or equal to the mean circulation term.

## Supplementary Material

Appendix 01 (PDF)

## Data Availability

Simulation data have been deposited in Zenodo (https://doi.org/10.5281/zenodo.18741956) (102).
