# Supplementary material for: Model evidence for distinct origins of glacial–interglacial and millennial signals in Greenland dust
Source: Proc Natl Acad Sci U S A. 2026 Mar 30;123(14):e2531908123. doi: 10.1073/pnas.2531908123 (PMC13056116; doi:10.1073/pnas.2531908123)
Supplement: Supplementary file 1 — Appendix 01 (PDF) [file pnas.2531908123.sapp.pdf]

Supporting Information for

Model evidence for distinct origins of glacial-interglacial and millennial signals in Greenland dust

Peter O. Hopcroft & Denis-Didier Rousseau

P.O. Hopcroft.
E-mail: p.hopcroft@bham.ac.uk

This PDF file includes:

Supporting text
Figs. S1 to S10
Tables S1 to S3
SI References

Supporting Information Text

Model-data evaluation

HadGEM2 is among the best performing models in CMIP5 (1) and has been widely used to study past and future climate processes (2–4). The LGM climate simulation with HadGEM2 has been evaluated against surface temperature and precipitation reconstructions and paleo-vegetation distributions (5) and is found to be comparable with other models used in PMIP. The precipitation response to the input of freshwater and AMOC slowdown (the FW1 and FW0.4 simulations, see figure S2) in HadGEM2 is compared with the multi-model mean of other comparable model simulations (6) in figure S7. Here multi-model mean across 17 simulations are averaged together but sub-selecting does not materially alter the spatial patterns shown. The patterns are normalised to improve comparability by dividing the global anomaly fields by the regional anomaly averaged over the North Atlantic (from 30-70°N). HadGEM2 and the multi-model mean show broadly similar features, particularly with significant drying signals over the Sahel, the Caribbean, mid-latitude Eurasia and the Sunda Shelf. HadGEM2 simulates a much more widespread drying over tropical South America and regions of precipitation increase south of the Himalayas.

In comparison with observationally-derived dust emissions Leung et al. (7), HadGEM2 successfully reproduces the main areas of dust activity as shown in figure 1 in the main text. Although the model includes a preferential source area this does not restrict emissions to the areas of observed dust, and so the agreement between HadGEM2 and Leung et al. (7) dataset indicates instead that the balance of physical climate and dynamic vegetation perform well in this model. For example, the model correctly predicts major dust emitting regions in the Sahara/Sahel, Arabia, Central Asia, Eastern Asia and others. The relative magnitude of these emitting regions is also in agreement with the Middle East/Central Asia and Sahel being the largest emitters in both cases, closely followed by the two divisions of the Sahara. However, due to a dry bias, the bare soil and dust emissions are overestimated in the eastern half of India and over Australia.

HadGEM2 has also been evaluated against a global compilation of pre-industrial and LGM dust flux reconstructions (8) and captures many of the main signals. The inclusion of glaciogenic dust follows the approach of (9) which is based on geological records of significant dust emissions sources that developed during the LGM. However, the strength of these sources regions (in terms of total dust emitted) and the particle distribution of these source areas is only indirectly constrained by observations. In our setup the relative strength of each of the glaciogenic source regions was optimised to reproduce the global LGM dust flux reconstructions as described before (10). However, the size distribution of these was not tuned. The glaciogenic sources cause a marked increase in particles in the size range 4-12 μm (diameter) deposited over Greenland (figure S8). In contrast Greenland ice-core records show that dustier intervals such as the LGM are instead characterised by an increase in finer particles in the range 0.6-2 μm (11). Therefore, the glaciogenic simulations most likely represent an upper limit on total dust loading over the high-latitudes during the LGM. At lower latitudes, HadGEM2's dust response to an AMOC slowdown has previously been evaluated against a compilation of high-resolution dust records (10).

One region of fairly strong emission in the LGM is the Yucatan Peninsula and the Caribbean where for the present-day, HadGEM2 is overly dry (12) and simulates too much bare soil as shown in figure 15 of ref. (13). This allows too much dust generation which is exacerbated under glacial conditions with stronger wind-speeds and reduced vegetation density. This type of precipitation bias is common over such narrow land-masses and is potentially related to the dependence on organised convection which is not well represented in most IPCC-class models (14) and is a subject of ongoing research.

Chemical/isotopic composition of North African dust

Modern dust samples from further south in North Africa e.g. the Sahel and surrounding areas show some similarities with Greenland glacial ice-core samples. Two areas might be of relevance to the present study. The Bodélé depression, the dustiest region in North Africa, shows very similar ^{87}Sr/^{86}Sr isotopic ratios and Nd (15) as Greenland ice-core samples for glacial stadial periods, as compared in figure S9. The chemical index of alteration (CIA) values measured for African dust including the Bodélé (16) are also relatively similar to Greenland glacial ice (17). A second region is along the western part of the Sahel. Here, samples from a transect over Niger/Benin/Togo (around 5-15°N) (18) have a very similar ϵHf and ϵNd as glacial Greenland ice-core values measured by Ujvari et al. (19) as shown in figure S10. Ujvari et al. (19) also argue that the hydrogen isotope compositions of the clay structural water (δD) can also provide further discrimination between these various regions. In that case, only South East Asian deserts, North Africa, and some areas of Eastern Central Europe can be considered as dominant sources for Greenland stadial phases when considering this and the elements/isotopes discussed above.

Table S1. **Dust cycle changes over the region 0 to 35°N by 80°W to 40°E.**

Time period	ΔEmissions (%)	ΔLoading (%)	ΔDeposition (%)
LGM-PreInd	94	91	-12.4
LGMglac-PreInd	95	92	66
LGMfw1-LGM	65	54	-14
LGMglacfw1-LGMglac	80	70	-6

Table S2. Summary of the main simulations analysed.

Run	Model	Time [kyr BP]	boundary conditions	Dust sources	Glaciogenic sources	Freshwater [Sv]	Reference
PreInd	HadGEM2-ES	0	CMIP5	Global	N	-	(8)
LGM	HadGEM2-ES	21	PMIP2	Global	N	-	(8)
LGMfw1	HadGEM2-ES	21	PMIP2	Global	N	1.0	(10)
LGMglac	HadGEM2-ES	21	PMIP2	Global	Y	-	(10)
LGMglac+fw1	HadGEM2-ES	21	PMIP2	Global	Y	1.0	(10)
LGMglac+fw0.4	HadGEM2-ES	21	PMIP2	Global	Y	0.4	(10)

Table S3. Differences in the total emission (across size bins 1-4) between the restricted-source and the global-source runs averaged over equivalent gridcells for the Northern Hemisphere.

Time period	Region	Restricted-sources - global-sources % error
**LGM-PreInd**		
	E Asia	-23
	C Asia/Arabia	-8
	Africa	-2
	Sahel	-8
	Europe	-18
**LGMfw1-LGM**		
	E Asia	2
	C Asia/Arabia	-40
	Africa	1
	Sahel	-43
**LGMglac-pi**		
	Africa	21
	Europe	31
**LGMglacf0.4-LGMglac**		
	Africa	8
	Europe	1

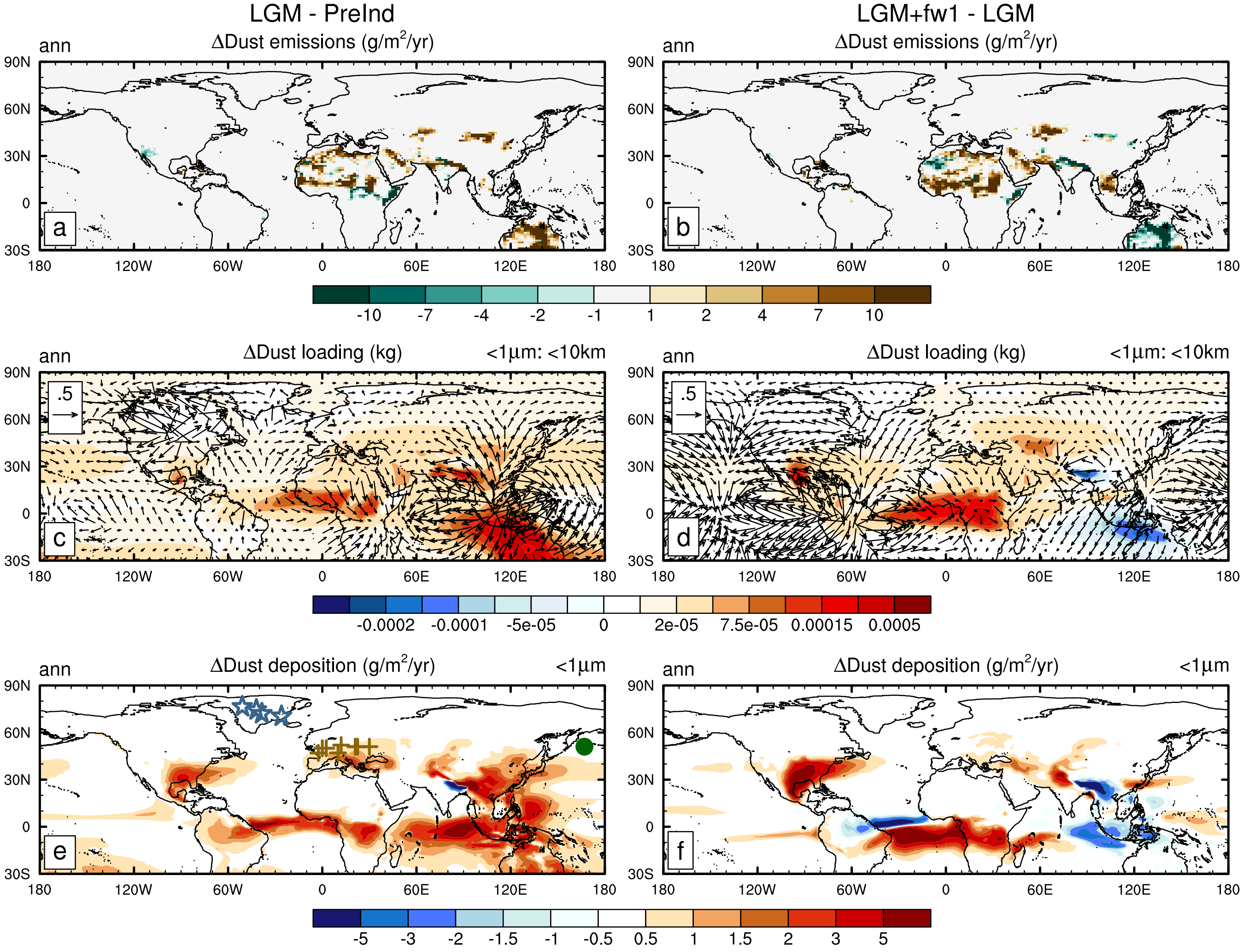

Fig. S1. Simulated response of the dust cycle LGM minus pre-Industrial (a,c,e) and LGMfw1 - LGM (b,d,f) for top row: dust emissions anomaly ($gm^{-2}yr^{-1}$), middle-row: dust column loading anomaly (up to 10 km) for fine particles (<1μm radius) (shading, %) overlaid with the anomaly of divergent wind vectors averaged over the middle troposphere (800-500 mbar, arrows), and bottom row: dust deposition anomaly for fine particles (<1μm radius) in %. The locations of Greenland ice-cores (blue stars) (20, 21), a North Pacific dust record (green circle) (22) and European loess cores (brown crosses) (23) are shown on panel e).

.

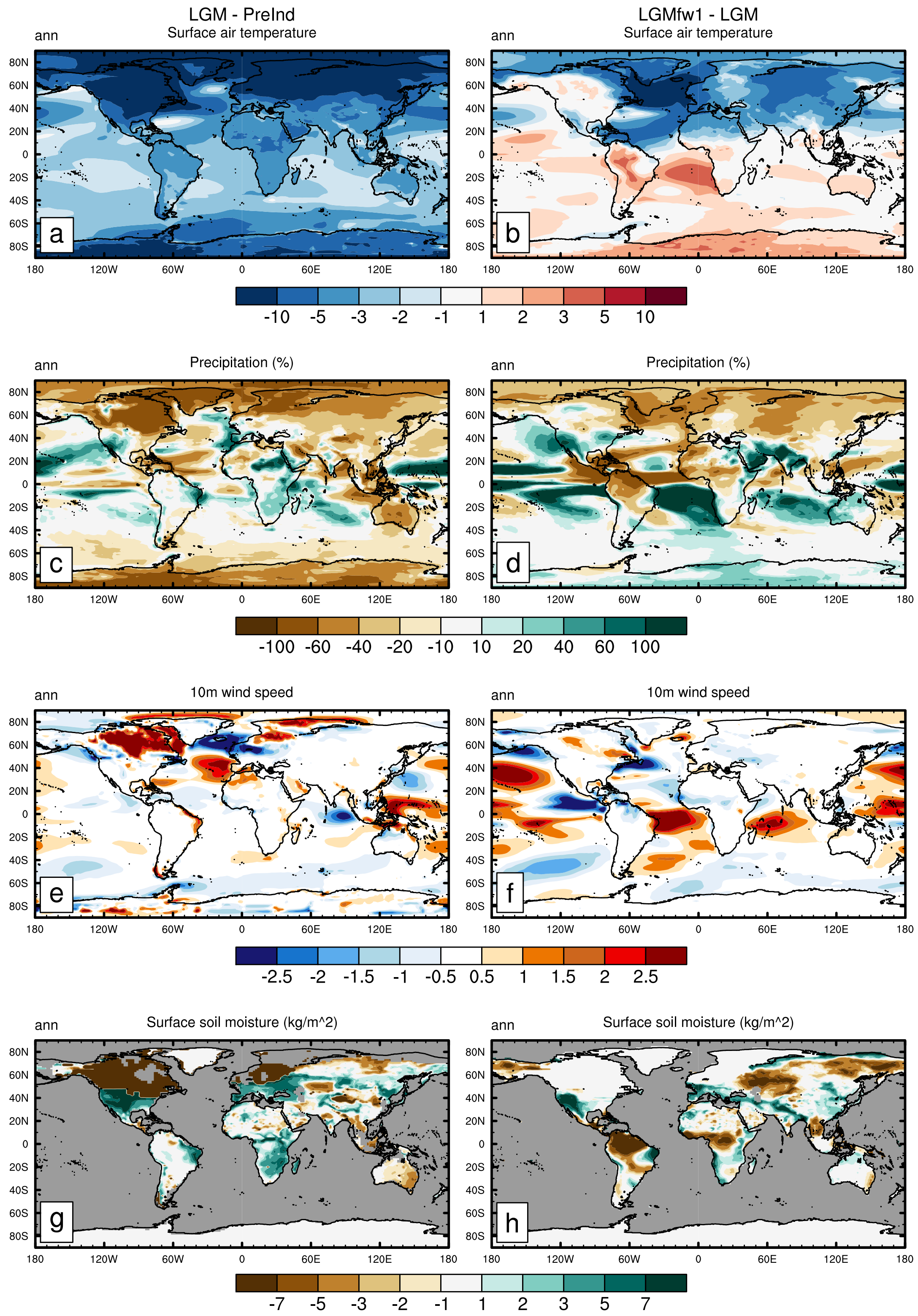

Fig. S2. Anomalies of surface air temperature (°C) (a,b), precipitation (%) (c,d), 10 m wind speed (ms^{-1}) (e,f) and soil moisture (kgm^{-2}) (e,f) for LGM minus pre-Industrial (left panels) and LGMfw1 minus LGM (right panels).

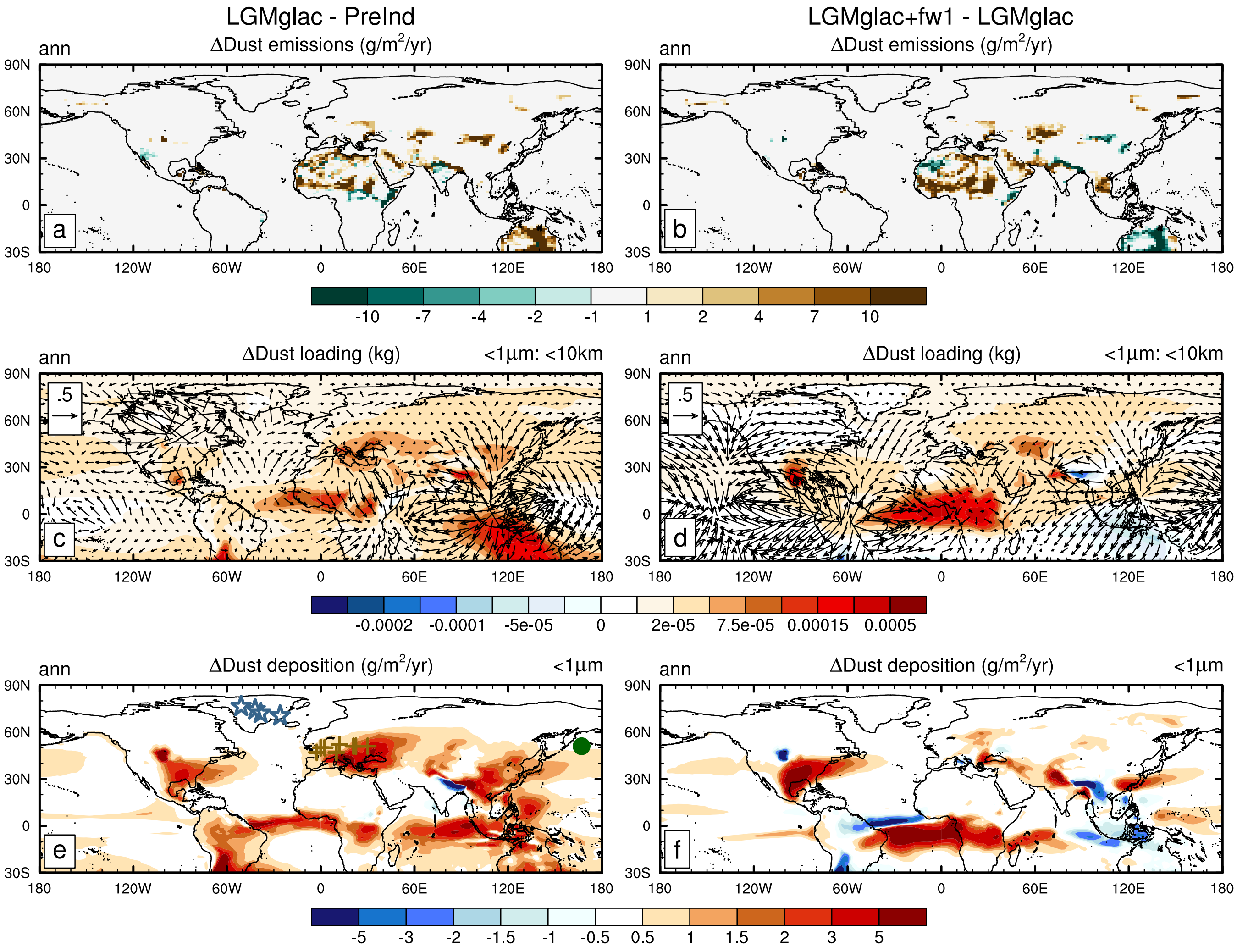

Fig. S3. As figure S1 but with glaciogenic dust sources included in the LGM and LGMfw1 simulations.

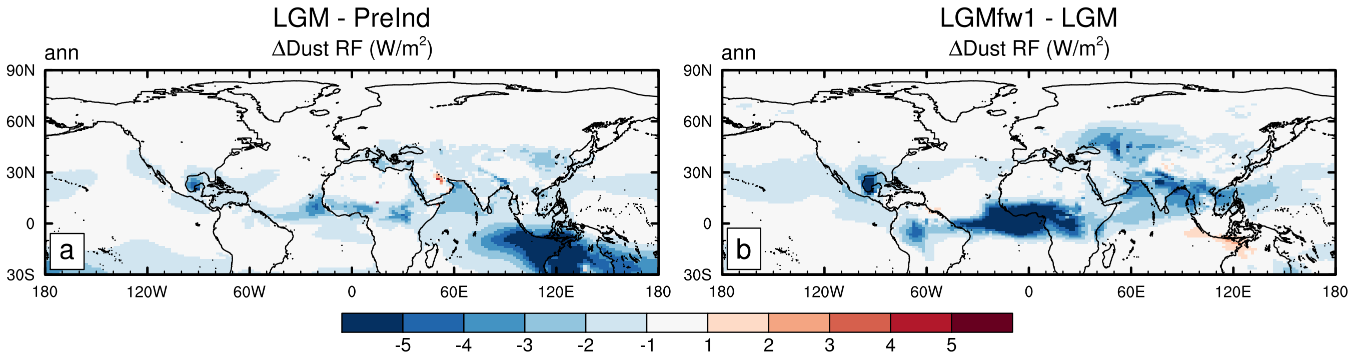

Fig. S4. Top-of-the-atmosphere net direct dust radiative forcing anomalies (Wm^{-2}) for a) LGM minus pre-Industrial and b) LGMfw1 minus LGM.

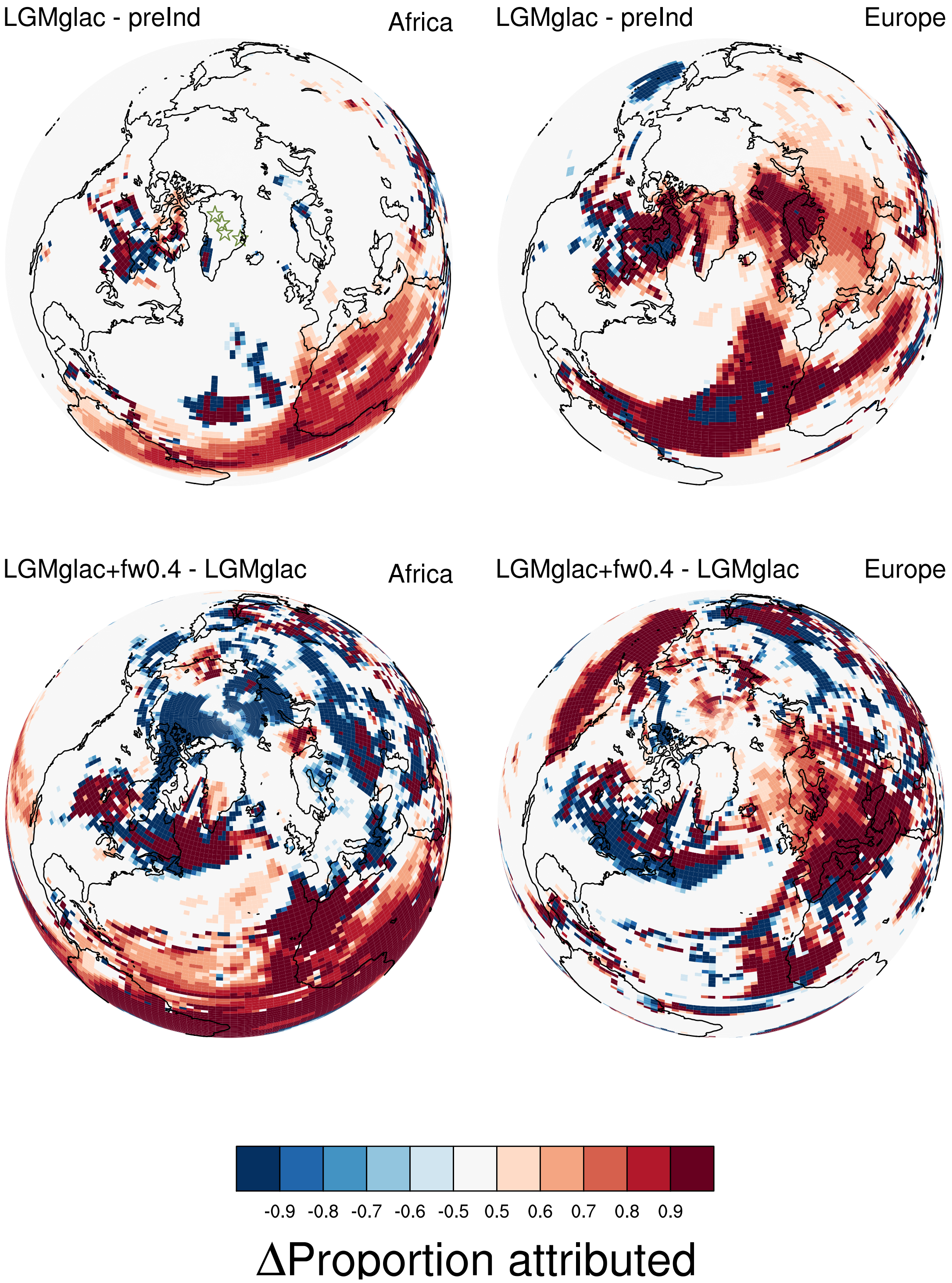

Fig. S5. As in figure 3 of the main text but comparing LGMglac with pre-Industrial and LGMglacfw0.4-LGMglac (where fw04 indicates a weaker freshwater forcing of only 0.4 Sv instead of 1.0 Sv).

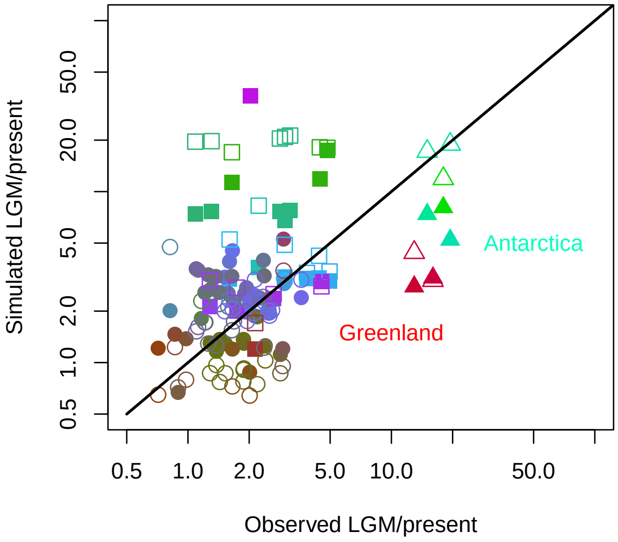

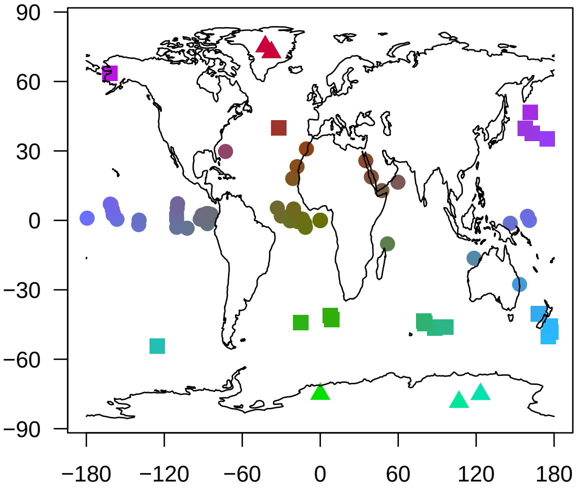

Fig. S6. The dust depositional flux LGM divided by pre-industrial. Left: observations from the Paleo±dust database (24) and ice-cores (25, 26) versus model output from HadGEM2-ES (filled symbols) and CESM (outline symbols) (27) both for particles $\leq 10\mu$m in diameter. Terrestrial records were excluded from the observations to focus on smaller particles that are subject to long-distance transport. The model simulations were averaged over ocean gridcells from 60°S-60°N and over Greenland and Antarctica (polewards of ±60°). Right: locations of all observational records used in this comparison.

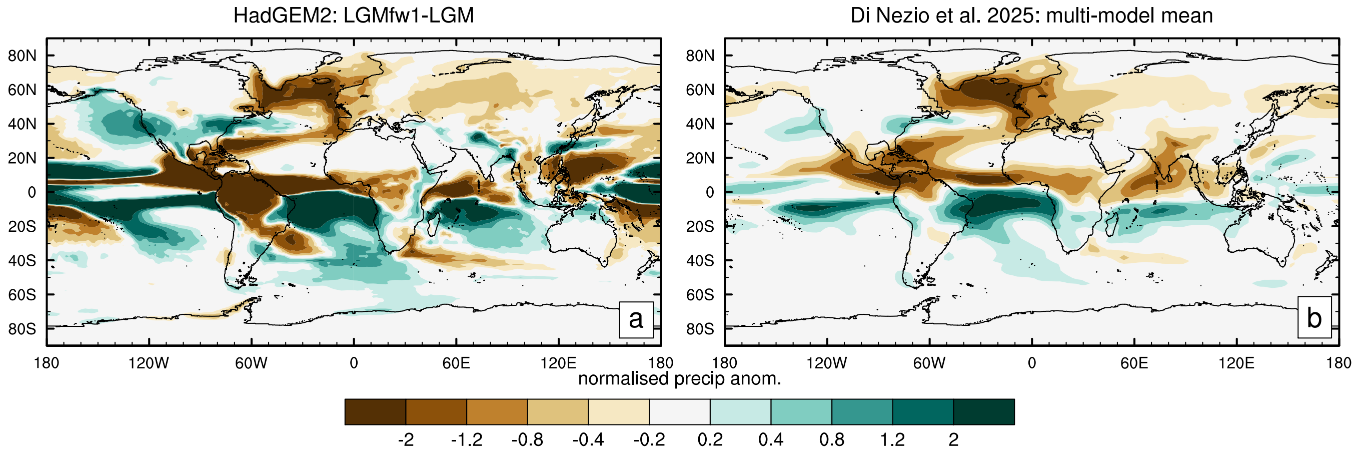

Fig. S7. Comparison of normalised precipitation response to AMOC slowdown in a) HadGEM2 and in b) the multi-model results from 17 different simulations using different climate models (6). The precipitation response was normalised by dividing the global field by the regional average anomaly calculated over the North Atlantic from 30-70°N.

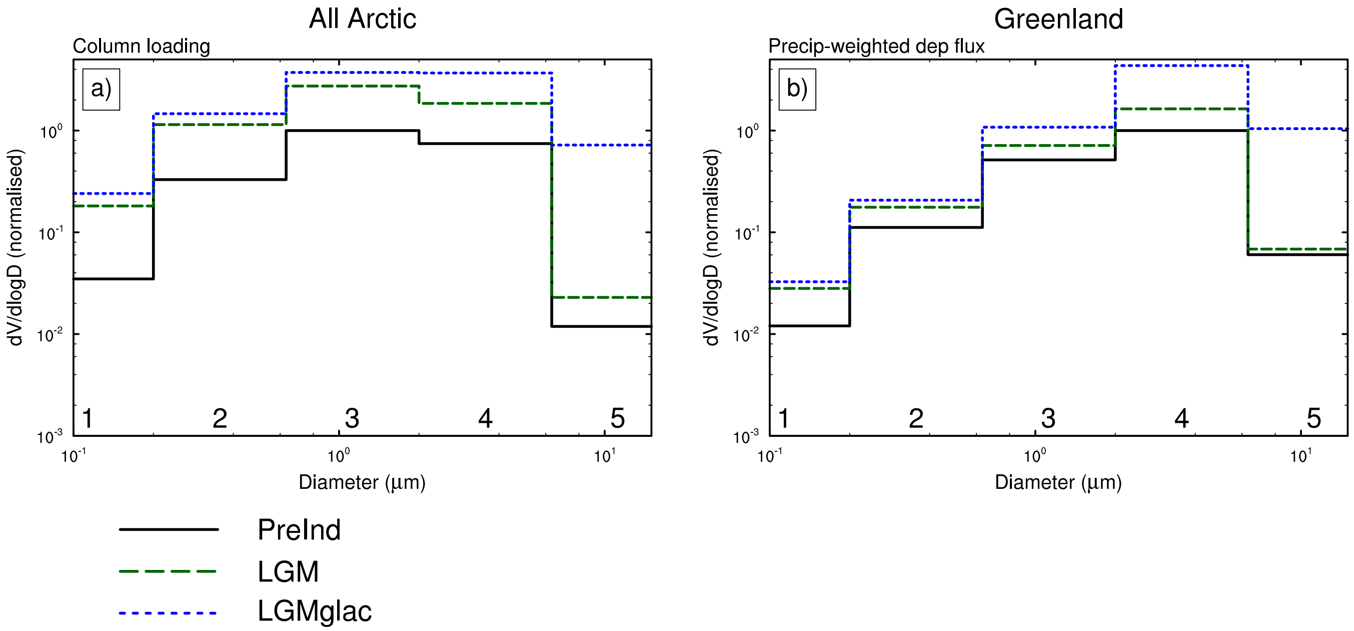

Fig. S8. Simulated dust particle size distribution for a) the total column loading over the Arctic >70N and b) annual-mean dust deposition flux over Greenland in the pre-Industrial, LGM and LGMglac simulations. The labels 1-5 indicate size ranges for the first five of the model's dust size bins. The largest size bin is not shown.

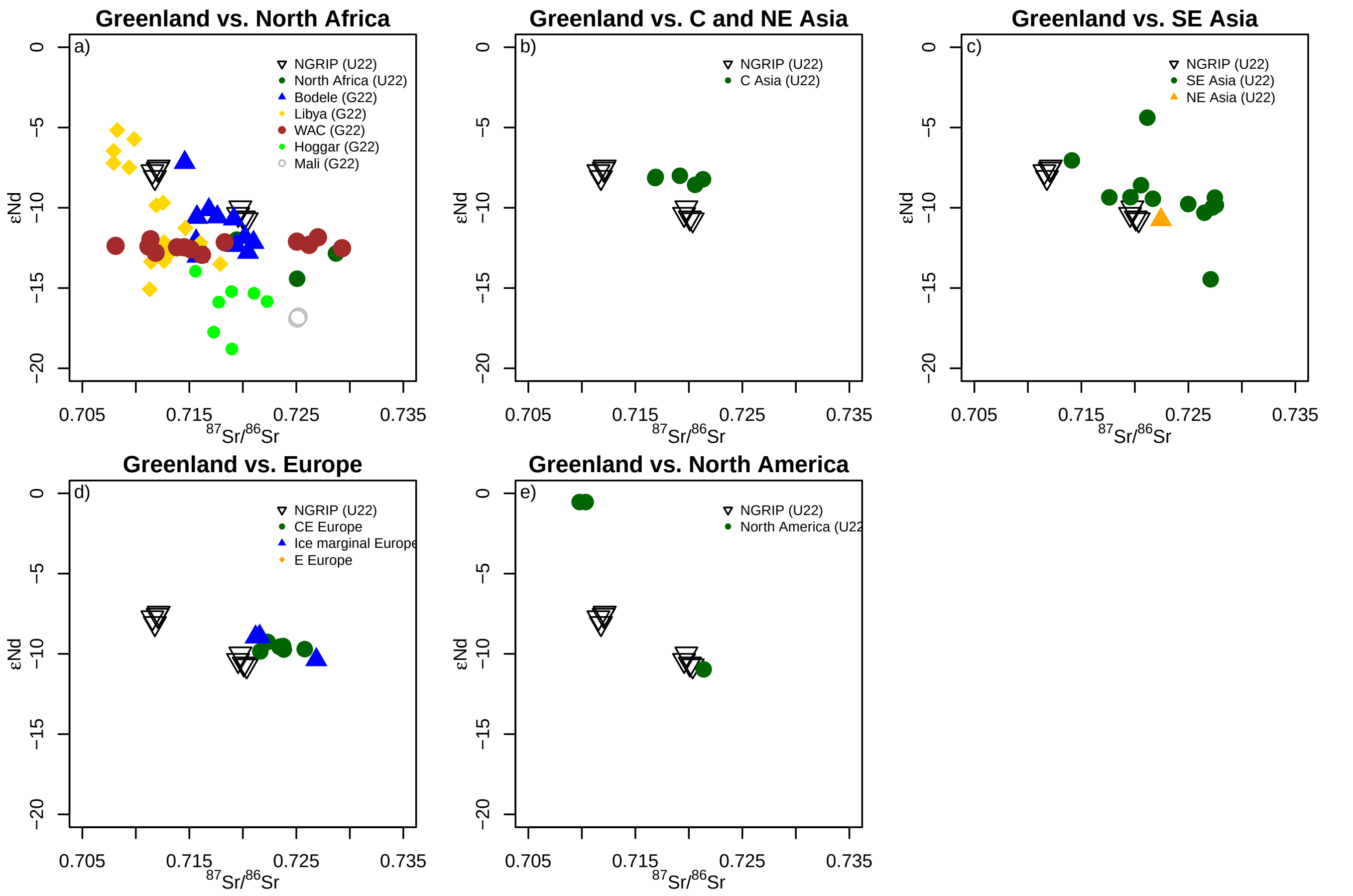

Fig. S9. Comparison of ice-core and sediment ϵNd and $^{8}7$Sr/$^{8}6$Sr isotope samples. NGRIP Greenland ice-core samples for the glacial stadial intervals from Ujvari et al. (U22) (19) compared with sediment samples from a) North Africa: from U22 and from Guinoisseau et al. (G22) (15); Comparisons for b) for Central and North East Asia, c) South East Asia, d) Europe and e) North America all as compiled by Ujvari et al. (U22) (19).

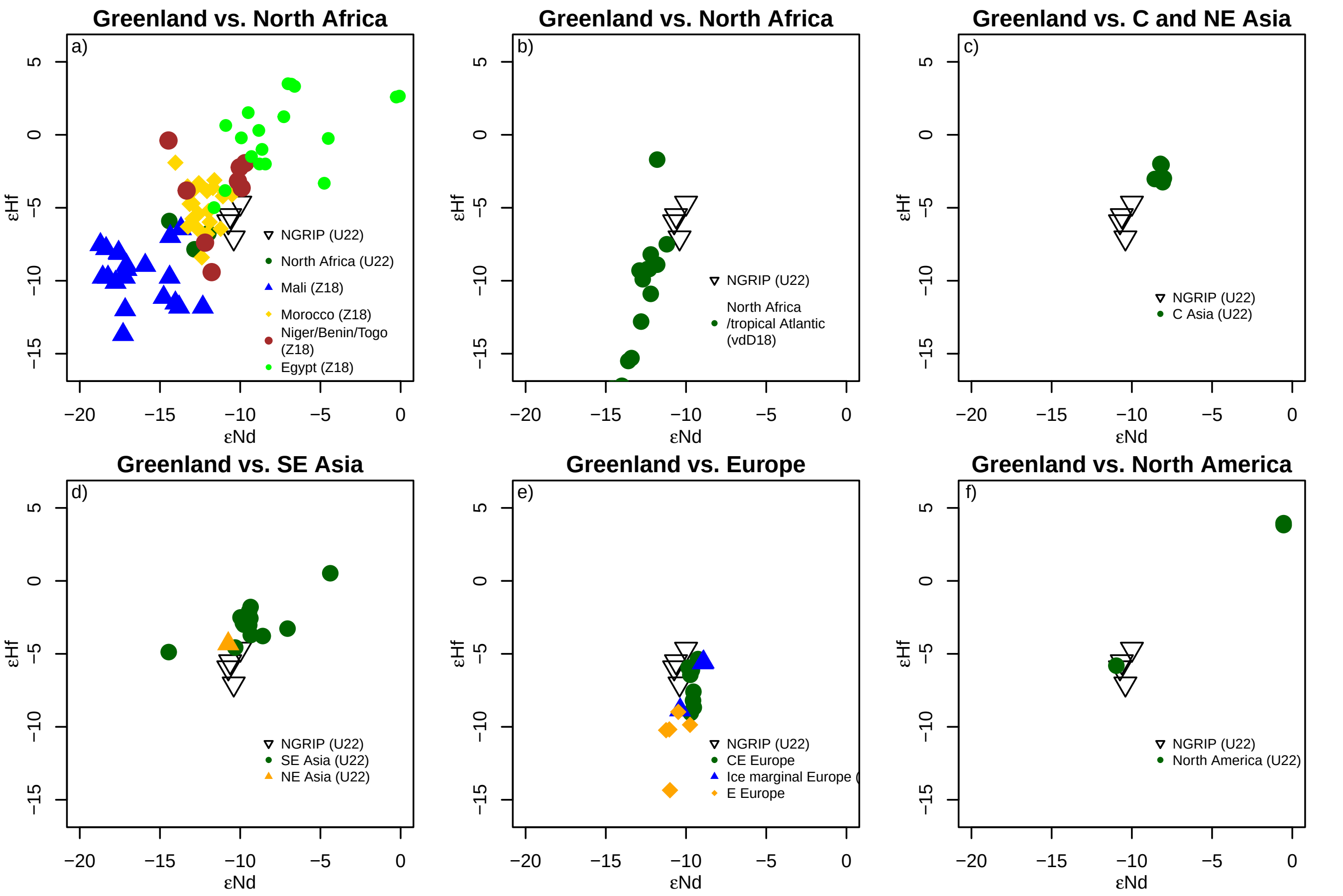

Fig. S10. Comparison of ice-core and sediment ϵNd and Hf samples. NGRIP Greenland ice-core samples for the glacial stadial intervals from Ujvari et al. (U22) (19) compared with sediment samples from a) North Africa: from U22 and from Zhao et al. (Z18) (18); North Africa and the tropical Atlantic from van der Does et al. (D18) (28). Comparisons for c) for Central and North East Asia, d) South East Asia, e) Europe and f) North America all as compiled by Ujvari et al. (U22) (19).

References

1. Valdes P, et al. (2017) The BRIDGE HadCM3 family of climate models: HadCM3@Bristol v1.0. *Geosci Model Dev* 10:3715–3743.
2. Booth B, Dunstone N, Halloran P, Andrews T, Bellouin N (2012) Aerosols implicated as a prime driver of twentieth-century North Atlantic climate variability. *Nature* 484(7393):228–232.
3. Caesar J, Palin E, Liddicoat S, , et al. (2013) Response of the HadGEM2 Earth System Model to Future Greenhouse Gas Emissions Pathways to the Year 2300. *J Clim* 26:3275–3284.
4. Tindall J, Haywood A (2020) Modelling the mid-Pliocene warm period using HadGEM2. *Glob Planetary Change* 186:103110.
5. Hopcroft P, Valdes P (2014) Last Glacial Maximum constraints on the Earth System model HadGEM2-ES. *Climate Dynamics* 45(5):1657–1672.
6. Di Nezio P, Shanahan T, Sun T, , et al. (2025) Tropical response to ocean circulation slowdown raises future drought risk. *Nature*.
7. Leung D, , et al. (2025) A global dust emission dataset for estimating dust radiative forcings in climate models. *Atmos Chem Phys* 25(2311-2331).
8. Hopcroft P, Valdes P, Woodward S, Joshi M (2015) Last glacial maximum radiative forcing from mineral dust aerosols in an Earth system model. *J Geophys Res* 120:8186–8205.
9. Albani S, et al. (2014) Improved dust representation in the Community Atmosphere Model. *Journal of Advances in Modeling Earth Systems* 6:541–570.
10. Hopcroft P, Pichat S, Valdes P, Kienast S (2023) Sensitivity of the tropical dust cycle to glacial abrupt climate changes. *Geophys Res Lett* 50(e2022GL101197).
11. Ruth U, Wagenbach D, Steffensen JP, Bigler M (2003) Continuous record of microparticle concentration and size distribution in the central Greenland NGRIP ice core during the last glacial period. *J Geophys Res* 108(D3, 4098).
12. Ryu JH, Hayhoe K (2014) Understanding the sources of Caribbean precipitation biases in CMIP3 and CMIP5 simulations. *Clim Dyn* 421:3233–3252.
13. Collins W, et al. (2011) Development and evaluation of an Earth-system model - HadGEM2. *Geosci Model Dev.* 4:1051–1075.
14. Neale R, Slingo J (2003) The Maritime Continent and Its Role in the Global Climate: A GCM Study. *J Clim* 16:834–848.
15. Guinoiseau D, Singh S, Galer S, , et al. (2022) Characterization of Saharan and Sahelian dust sources based on geochemical and radiogenic isotope signatures. *Quat Sci Rev* 293(107729).
16. Moreno T, Querol X, Castillo S, , et al. (2006) Geochemical variations in aeolian mineral particles from the Sahara–Sahel Dust Corridor. *Chemosphere* 65:261–270.
17. Ro S, Park J, Yoo H, , et al. (2024) Millennial-scale variability of Greenland dust provenance during the last glacial maximum as determined by single particle analysis. *Scientific Reports* 14(2040).
18. Zhao W, Balsam W, Williams E, , et al. (2018) Sr-Nd-Hf isotopic fingerprinting of transatlantic dust derived from North Africa. *Earth Planet Sci Lett* 46:23–31.
19. Ujvari G, Klotzli U, Stevens T, Svensson A, , et al. (2022) Greenland ice core record of last glacial dust sources and atmospheric circulation. *J Geophys Res* 127(15):e2022JD036597.
20. Ruth U, et al. (2007) Ice core evidence for a very tight link between North Atlantic and east Asian glacial climate. *Geophys Res Lett* 34(3).
21. Schupbach S, Fischer H, Bigler M (2018) Greenland records of aerosol source and atmospheric lifetime changes from the Eemian to the Holocene. *Nature Communications* 9(1476).
22. Serno S, et al. (2015) Comparing dust flux records from the Subarctic North Pacific and Greenland: Implications for atmospheric transport to Greenland and for the application of dust as a chronostratigraphic tool. *Paleoceanography* 30.
23. Rousseau DD, , et al. (2014) European glacial dust deposits: Geochemical constraints on atmospheric dust cycle modeling. *Geophys. Res. Lett.* 41:7666–7674.
24. Cosentino N, et al. (2024) Paleo±dust: Quantifying uncertainty in paleo-dust deposition across archive types. *Earth System Scientific Data* 16(2):941–959.
25. Fischer H, , et al. (2007) Glacial/interglacial changes in mineral dust and sea-salt records in polar ice cores: Sources, transport, and deposition. *Rev. Geophysics* 45(RG1002).
26. Wegner A, Fischer H, Delmonte B, , et al. (2015) The role of seasonality of mineral dust concentration and size on glacial/interglacial dust changes in the EPICA Dronning Maud Land ice core. *J. Geophys. Res. Atmos.,* 120:9916–9931.
27. Albani S, et al. (2016) Paleodust variability since the Last Glacial Maximum and implications for iron inputs to the ocean. *Geophys Res Lett* 43.
28. van der Does M, Pourmand A, Sharifi A, Stuut JB (2018) North African mineral dust across the tropical Atlantic Ocean: Insights from dust particle size, radiogenic Sr-Nd-Hf isotopes and rare earth elements (REE). *Aeolian Research* 33:106–116.
